# Constipation

**Published:** 1883-04

**Authors:** 


### CONSTIPATION.

CONSTIPATION is the beginning of many diseases. It is the most prevalent of all affections among those not accustomed to out-door activities. It frequently commences in infancy through the neglect or ignorance of parents; and the health sometimes becomes permanently impaired, before the cause is discovered by the physician.

There should be at least one free and natural movement of the bowels every day, and when that is not the case, all proper means should be promptly employed to bring it about. Nature intends that the waste material, after digestion is completed, shall be passed out of the system within a certain time, but if that time is exceeded it commences to be absorbed, thus the blood is poisoned and the vital force is impaired; hence the body becomes an easy prey to disease.

Dyspepsia is generally the first diseased condition caused by constipation. The liver soon becomes involved as a result of indigestion, then the kidneys. It is evident that a long continued derangement of either of these important organs must result most unfortunately. All experience proves that habitual constipation is a very unsafe condition of the system, and one liable at any time to develop incurable diseases.

Various plans have been devised for the cure of this distressing complaint; but we do not believe in restricting the treatment to any one remedy. To secure success various methods must be employed, and employed persistently. Some will after a while lose their effect, and others must be substituted ; no quarter should be shown until this great enemy to health is overcome. The habit of taking purgative medicines to relieve the bowels often increases the trouble ; that is, the system becomes accustomed to this remedy and there is no relief without it ; the remedy debilitates, and it becomes only a question of time how long the treatment can be borne.

As in these cases there is always a torpid liver, we should commence the treatment with a mild cathartic—as two or three liver pills ; and then pay especial attention to the diet. Bread made from crushed wheat or oat meal should be used ; we should not restrict the patient as to other foods, except as to quantity. He should eat enough, but not overload the stomach. A tumbler of cold water with a teaspoonful of table salt dissolved in it and drank every morning half an hour before breakfast often acts like magic in restoring the bowels to their natural condition. There are many cases of obstinate constipation, where the whole trouble exists in the lower part of the rectum, by impacting of fecal matter, due to feeble action of the muscles, and to a congested and dry condition of the mucous membrane at that point. We have never found a remedy that so promptly relieved this form of constipation as *Nelaton's Suppository*, the advertisement and the formula of which may be found in this Journal. This treatment alone is sometimes sufficient to cure such cases ; and where the trouble is more general, the suppository will be found a most valuable addition to the list of remedies.

Regular and vigorous out-door exercise is all important. Kneading the bowels with the hands has been recommended ; also, the drinking of water frequently, to which we should always add a little table salt.

The frequent use of a syringe should be avoided, for much the same reason that cathartics ought to be avoided. No harsh or very active treatment is required in these cases ; but mild remedies may be employed persistently ; in fact, they should never be remitted until the bowels become regular and the health is restored. We believe that a majority of cases are curable. We know of one case of great severity that lasted twenty-two years, and was then cured, although the general health has never been fully restored.

